# Pregnancy and Childbirth in Neurodivergent Women: Shift Towards Personalized Maternity Care

**DOI:** 10.3390/jpm15110557

**Published:** 2025-11-17

**Authors:** Anna M. Avdeeva, Mariia A. Parfenenko, Elena V. Bryzgalina, Kamilla T. Muminova, Zulfiya S. Khodzhaeva

**Affiliations:** 1 High Risk Pregnancy Department, National Medical Research Center for Obstetrics, Gynecology, and Perinatology Named After Academician V.I. Kulakov of the Ministry of Health of the Russian Federation, Oparina Str. 4, 117997 Moscow, Russia; k_muminova@oparina4.ru (K.T.M.); z_khodzhaeva@oparina4.ru (Z.S.K.); 2 Faculty of Medicine, M. V. Lomonosov Moscow State University, Leninskie Gory, 1, Building 31, 119991 Moscow, Russia; masha.parfenenko@student.msu.ru (M.A.P.); bryzgalinaev@my.msu.ru (E.V.B.); 3 Veltischev Research and Clinical Institute for Pediatrics and Pediatric Surgery, Pirogov Russian National Research Medical University, 125412 Moscow, Russia; 4 Faculty of Philosophy, M. V. Lomonosov Moscow State University, GSP-1, Lomonosovsky Prospekt, 27-4, 119991 Moscow, Russia

**Keywords:** neurodevelopmental disorders, neurodivergent women, neurodiversity-informed perinatal care, personalized perinatal care, ethical standards of care, pregnancy and childbirth, obstetric ethics, maternal autonomy, informed consent in obstetrics

## Abstract

**Introduction:** Neurodevelopmental disorders (NDs), including autism spectrum disorder and related conditions, are increasingly recognized among women of reproductive age, yet their unique needs during pregnancy and childbirth remain poorly studied. Communication differences, sensory sensitivities, and co-occurring psychiatric conditions may complicate maternity care, leading to higher risks of adverse outcomes and ethical challenges in clinical practice. This study aimed to examine pregnancy complications, delivery outcomes, and postpartum characteristics in women with NDs, compared with a control group, and to identify specific barriers in perinatal care. **Methods:** A retrospective observational study was conducted at the National Medical Research Center for Obstetrics, Gynecology and Perinatology, Moscow, including 18 pregnant women with confirmed NDs and 21 matched controls with uncomplicated pregnancies. Data were extracted from medical records and included demographic parameters, pregnancy course, complications, labor management, neonatal outcomes, and documented communication or ethical issues. Comparative analyses were performed using chi-square or Fisher’s exact tests for categorical variables and Student’s *t*-test or Mann–Whitney U test for continuous variables. **Results:** Pregnant women with NDs had significantly higher rates of pelvic girdle pain (66.7% vs. 23.8%, *p* = 0.01), vaginal bleeding (44.4% vs. 14.3%, *p* = 0.04), anxiety (61.1% vs. 19.0%, *p* = 0.007), and depression (50.0% vs. 14.3%, *p* = 0.02) compared with controls. Persistent daily nausea was also more common (50.0% vs. 14.3%, *p* = 0.03). Attendance of prenatal physician visits was lower in the ND group (66.7% vs. 95.2%, *p* = 0.02). Cesarean delivery occurred in 83.3% of ND women versus 23.8% of controls (*p* < 0.001), with psychiatric recommendations often cited as the indication. Breastfeeding was declined in 94.4% of ND cases versus 4.8% of controls. Labor duration was prolonged, and neonatal anthropometrics were lower in the ND group. Communication difficulties were documented in 83.3% of ND participants, and postpartum depressive symptoms were identified in 77.8%. **Conclusions:** Pregnant women with NDs face a multidimensional vulnerability in maternity care, including higher frequencies of pain, bleeding, nausea, anxiety, and depression, prolonged labor, markedly increased cesarean rates, reduced breastfeeding initiation, and smaller neonatal anthropometrics. Frequent communication barriers, guardian decision-making, and postpartum separation further complicate care. These findings underscore the necessity of neurodiversity-informed, individualized perinatal strategies, integrating sensory accommodations, trauma-informed communication, and proactive mental health support to improve both clinical outcomes and patient experiences.

## 1. Introduction

Neurodevelopmental disorders (NDs) are a group of conditions caused by abnormal development of the nervous system and characterized by differences in cognition, communication, sensory processing, and behavioral patterns. NDs include autism spectrum disorder (ASDs), attention-deficit/hyperactivity disorder (ADHD), learning disabilities, and intellectual disability. While historically perceived as a male-dominated conditions, growing recognition reveals a significant population of women who have been systematically overlooked [1,2,3]. This invisibility, stemming from diagnostic overshadowing, gender-biased diagnostic criteria, and sophisticated camouflaging strategies, has profound implications for their healthcare experiences. As these women enter the maternity care system, their unique needs necessitate deliberate and informed adaptations within the labor and delivery (L&D) environment to ensure equitable, trauma-informed, and high-quality care.

Interindividual with NDs often have communication differences that may hinder effective interaction with medical workers, especially in the stressful environment of L&D. Additionally, differences in sensory processing (abnormal pain processing, aversion to certain forms of physical touch and hypersensitivity to sensory stimuli) make women with NDs especially vulnerable to the stressors associated with childbirth, leading to additional overwhelm and discomfort. That, coupled with the necessary but intrusive procedures—such as cervical examinations, fetal monitoring, and intravenous lines—can trigger sensory overload or shutdown, potentially hindering labor progress and complicating clinical management. Therefore, women with NDs are especially sensitive to the sensory and social intensity of perinatal care [4,5,6].

Women with NDs represent a particularly challenging population in obstetric practice [7,8]. The diagnosis in women is often delayed or overlooked, as clinical manifestations may be subtle or masked, and social stereotypes frequently contribute to underdiagnosis. Consequently, many women enter pregnancy without a confirmed diagnosis or with insufficient awareness of their condition among healthcare providers. This creates additional difficulties in establishing effective communication, ensuring adherence to medical recommendations, and planning individualized obstetric care [9].

Women with NDs often enter pregnancy having faced a lifetime of healthcare disparities. While individuals with NDs tend to receive lower quality of medical care, due to misdiagnosis and late recognition, coupled with a tendency for healthcare providers to misinterpret neurodivergent traits in women specifically, frequently leading to miscommunication with medical professionally, resulting in poorer care quality and a high prevalence of negative or traumatic medical encounters [10,11,12]. These prior experiences can establish a foundation of mistrust and anxiety, significantly complicating the patient–provider relationship during the vulnerable process of childbirth.

Co-occurring mental health conditions, specifically anxiety and stress disorders are highly prevalent among individuals with NDs. The inherent uncertainties of labor, the loss of personal autonomy, and the need for constant communication can acutely exacerbate these conditions. This elevated anxiety can impair an individual’s ability to effectively articulate their needs, understand complex medical information, or provide consent during rapidly evolving situations, thereby negatively impacting their sense of wellbeing and agency during birth [13,14].

Finally, beyond psychosocial challenges, women with NDs face increased risks of complications related to pregnancy and birth. Research indicates a higher prevalence of comorbid conditions such as epilepsy, connective tissue disorders, and gastrointestinal issues, all of which can contribute to a higher incidence of complications, including preterm birth and pre-eclampsia [15,16,17,18]. This complex medical profile the need for heightened clinical vigilance and an integrated care approach.

Research on the pregnancy experiences of people with NDs remains limited [19]. At the same time, a growing number of studies have examined pregnancy among individuals with various other disabilities, including mental health conditions and intellectual disabilities [20]. Insights from such studies may help to better understand the experiences of neurodivergent people as intellectual disability and mental health challenges frequently co-occur with NDs and may involve similar difficulties [21].

Many healthcare providers and specialists in the field of obstetrics have limited knowledge and training when it comes to managing the needs of women with NDs [9]. This lack of awareness not only complicates the provision of adequate medical care but also contributes to communication barriers, reduced accessibility of services, and a higher risk of overlooking important clinical needs. Addressing this gap is therefore essential for ensuring equitable and patient-centered obstetric care.

Despite being particularly vulnerable in the L&D ward setting, women with NDs are a frequently overlooked population [5]. The current standard protocols for perinatal care are insufficient for such women. A research-based protocol, tailored to meet the needs of women with NDs is needed to improve both clinical outcomes and the subjective birth experience [22].

There is a scarcity of previous research on pregnancy and childbirth experiences in neurodivergent women, particularly in clinical settings. This limited body of literature restricts the ability to contextualize findings, draw robust comparisons, and formulate evidence-based recommendations. Moreover, existing studies often focus on mental health or neonatal outcomes in isolation, without integrating psychosocial, physical, and healthcare experience dimensions simultaneously.

A comprehensive assessment of pregnant women with NDs is essential, as insufficient awareness of healthcare personnel regarding the specific needs of this group often leads to misunderstandings and conflicts in clinical practice [23]. Therefore, the present study was designed to analyze the course of pregnancy and labor management in women with NDs. Based on these findings, we aimed to develop recommendations for healthcare professionals that incorporate bioethical principles, effective communication strategies, and tailored approaches to perinatal care. This approach may contribute to more individualized perinatal care strategies for women with NDs.

## 2. Materials and Methods

This retrospective observational study was conducted at the National Medical Research Center for Obstetrics, Gynecology and Perinatology named after Academician V.I. Kulakov, Moscow, Russia. The study is reported in accordance with the Strengthening the Reporting of Observational Studies in Epidemiology (STROBE) guidelines.

The study group included 21 women with confirmed neurodevelopmental disorders, primarily autism spectrum disorder and related conditions, who received antenatal care and delivered at our Center between 2015 and 2025. Inclusion criteria were: maternal age 18–45 years, singleton pregnancy, confirmed diagnosis of a neurodevelopmental disorder according to ICD-11 (or equivalent earlier classifications), and availability of complete medical records. The decision to examine women with NDs as a singular group was made due to high comorbidity of all NDs, as well as heterogeny in their clinical presentation [24,25,26]. In essence, women with multiple NDs and/or mental disorders are more prevalent, than those with a single condition, and often have shared experiences despite some variation in their diagnoses. The small sample size reflects diagnostic limitations rather than narrow inclusion criteria, since obtaining an official diagnosis of neurodevelopmental disorders in adult women in Russia is a complex, multi-step process requiring evaluation by several specialists and final confirmation by a psychiatrist. To maintain scientific accuracy, only women with confirmed diagnoses were included. Additionally, due to gender biases and unequal access to diagnostic services, some comorbid NDs and/or mental health conditions may remain unidentified in women. Exclusion criteria were multiple pregnancy, major congenital anomalies, oncological disease, history of organ transplantation, and decompensated systemic conditions.

The control group consisted of women with uncomplicated pregnancies who were matched to the study group by maternal age, parity and gestational age (pairwise matching). Controls were selected from women who received antenatal and intrapartum care at the same center and during the same study period.

In this study, the term “uncomplicated pregnancy” refers to pregnancies without pre-existing maternal chronic diseases or significant obstetric complications. Specifically, women in the control group had no history or diagnosis of any kind of psychiatric conditions nor neurodevelopmental disorders.

To avoid selection bias, matching of control participants was performed before reviewing pregnancy outcomes, based on antenatal data available at the time of delivery planning (maternal age, parity, and gestational age). The obstetric outcomes were analyzed only after the matching process was completed.

Clinical and anamnestic data were extracted from medical records, including demographic information, obstetric history, course of pregnancy, complications, labor management, and delivery outcomes. Pregnancy complications were defined according to standard obstetric criteria (e.g., preeclampsia, gestational diabetes, preterm birth < 37 weeks, operative delivery for maternal or fetal indications). Perinatal complications included low birthweight (<2500 g), Apgar score < 7 at 5 min, and admission to the neonatal intensive care unit (NICU).

In addition to clinical outcomes, special attention was paid to ethical challenges documented in the medical records, including issues related to informed consent, participation of legal guardians or relatives in decision-making, refusal of therapy, and communication difficulties between healthcare providers and patients. Ethical analysis was performed in line with the principles of autonomy, beneficence, non-maleficence, and justice, as outlined in the Declaration of Helsinki and the Belmont Report.

Quantitative data were summarized using descriptive statistics (means, standard deviations, medians, and interquartile ranges). Comparative analysis between groups was performed using the chi-square test or Fisher’s exact test for categorical variables and Student’s *t*-test or the Mann–Whitney U test for continuous variables, depending on data distribution. A *p*-value < 0.05 was considered statistically significant. All analyses were performed using Prism (GraphPad Prism v 10.6.1).

Comparative analysis between groups was performed using chi-square or Fisher’s exact tests for categorical variables and Student’s *t*-test or Mann–Whitney U test for continuous variables. To minimize the effect of confounding, logistic regression models were used to calculate adjusted odds ratios (aORs), controlling for maternal age, parity, and gestational age at delivery. Adjusted *p*-values were derived using the Benjamini–Hochberg false discovery rate (FDR) correction.

All data were anonymized prior to analysis. The study protocol was approved by the Ethics Committee of the National Medical Research Center for Obstetrics, Gynecology and Perinatology, and the research was conducted in accordance with the principles of the Declaration of Helsinki.

## 3. Results

A total of 21 neurodivergent women and 24 women in the control group were initially enrolled. Three participants from the neurodivergent group were subsequently excluded for not meeting eligibility criteria, leaving 18 cases and 21 controls for analysis.

### 3.1. Baseline Characteristics

The mean age of women in the neurodivergent group was 32.5 ± 5.7 years (range: 21–43), compared with 30.7 ± 4.1 years (range: 24–41) in the control group. The mean height was 165.4 ± 6.2 cm in the neurodivergent group and 167.8 ± 6.5 cm in the control group. Mean body mass index (BMI) was 26.8 ± 5.1 kg/m^2^ versus 24.5 ± 4.8 kg/m^2^, respectively. No significant differences were observed for age or height, although BMI indicated a trend toward higher body weight in the neurodivergent group.

### 3.2. Obstetric History

Among neurodivergent women (*n* = 18), 7 (38.9%) were primiparous, 6 (32.0%) had parity 2, and 5 (29.1%) had parity ≥ 3. In the control group (*n* = 21), 8 (40.0%) were primiparous, 7 (33.8%) had parity 2, and 6 (26.2%) had parity ≥ 3. The distribution of parity did not differ significantly between groups (χ^2^(2) = 0.004, *p* = 0.998).

### 3.3. Pregnancy Complications

Complications such as threatened preterm labor, hypertensive disorders, anemia, and toxicosis were reported in 83.3% of the neurodivergent group and 70.0% of controls (*p* = 0.4543). Pelvic girdle pain and vaginal bleeding occurred significantly more often among neurodivergent women (Table 1). Symptoms of anxiety were reported by 11 of 18 (61.1%) cases versus 4 of 21 (19.0%) controls (*p* = 0.007), while depressive symptoms were reported by 9 of 18 (50.0%) versus 3 of 21 (14.3%), respectively (*p* = 0.02) (Table 1). Persistent daily nausea (“all day, every day”) was described by 9 of 18 women (50.0%) in the neurodivergent group and 3 of 21 women (14.3%) in the control group (Fisher’s exact test, *p* = 0.03) (participants reported nausea frequency only for the gestational period in which the symptom was present) (Table 2).

### 3.4. Attendance of Prenatal Appointments

16 of 18 women (88.9%) in the neurodivergent group and 20 of 21 women (95.2%) in the control group attended all scheduled ultrasound examinations (Fisher’s exact test, *p* = 0.59). Physician appointments were fully attended by 12 of 18 neurodivergent participants (66.7%) compared with 20 of 21 controls (95.2%) (Fisher’s exact test, *p* = 0.02) (Table 3).

### 3.5. Delivery Outcomes

The mean gestational age at delivery was 37.7 ± 1.8 weeks in the neurodivergent group and 38.5 ± 1.2 weeks in controls. Refusal of minor medical interventions—such as intravenous catheter placement, intravenous infusions, amniotomy, or episiotomy—was significantly more frequent among cases (41.9% vs. 4.8%, *p* = 0.004). These refusals typically concerned routine obstetric procedures rather than major interventions. Importantly, refusal of cesarean delivery was not considered in this category, as almost all women ultimately underwent surgical delivery when clinically indicated. Operative delivery predominated in the neurodivergent group, with 15 of 18 women (83.3%) undergoing cesarean section compared with 5 of 21 controls (23.8%) (*p* < 0.001). Indications for cesarean delivery in all cases followed traditional obstetric criteria in accordance with the FIGO Good Practice Recommendations for Cesarean Delivery [27]. Psychiatric recommendations were documented as the indication in 10 of these cases. In three cases, the decision for cesarean delivery was made at the discretion of the attending obstetricians, as they considered it the most appropriate management strategy due to altered mental status of the patient. In the remaining two cases, cesarean section was indicated due to obstetric factors, namely a history of previous cesarean delivery and breech presentation in a fetus with high estimated weight of the fetus.

Breastfeeding was declined by 17 of 18 women (94.4%) in the neurodivergent group, including 6 refusals following neonatologist consultation, whereas breastfeeding initiation was reported in 20 of 21 (95.2%) controls.

Among the three neurodivergent women who delivered vaginally, the mean duration of the first stage of labor was 19.4 ± 4.1 h, the second stage 2.8 ± 0.9 h, and the third stage 0.12 ± 0.05 h. In the control group (*n* = 16 vaginal deliveries), the corresponding durations were 11.2 ± 3.5 h, 1.3 ± 0.6 h, and 0.08 ± 0.04 h, indicating longer first and second stages among neurodivergent women.

### 3.6. Neonatal Outcomes

Mean birth weight was 2920 ± 410 g in the neurodivergent group versus 3340 ± 380 g in controls, and mean birth length was 48.1 ± 2.5 cm versus 50.4 ± 2.8 cm, respectively. When adjusted for gestational age using standardized fetal growth charts (INTERGROWTH-21st), these mean birth weights correspond approximately to the 30th percentile in the neurodivergent group (mean GA 37.7 ± 1.8 w) and to the 50th percentile in controls (mean GA 38.5 ± 1.2 w), indicating that infants in both groups were, on average, appropriate for gestational age (AGA).

### 3.7. Postpartum Chatacteristics

Medical decisions made by a guardian or trustee were recorded in 5 of 18 cases (27.1%) and in none of the controls (*p* = 0.051). Postpartum separation of mother and newborn occurred in 10 of 18 (55.6%) neurodivergent women, with formula feeding initiated under medical supervision in three cases. Documented communication difficulties were observed in 15 of 18 women (83.3%) during pregnancy and labor. Seven women (38.9%) received maintenance therapy with antipsychotic or antidepressant medications during pregnancy. Postpartum depressive symptoms were identified in 14 of 18 (77.8%) cases based on medical records.

## 4. Discussion

This study illuminates significant differences in the pregnancy and labor experiences of neurodivergent women, moving beyond a mere catalog of clinical outcomes to highlight a critical imperative: the need for a systemic shift toward neurodiversity-informed, trauma-conscious maternity care. While we identified several key clinical disparities—including a significantly elevated rate of cesarean section, prolonged labor, and lower rates of breastfeeding initiation in the neurodivergent group—the overarching narrative is not one of inherent pathology but of a healthcare environment ill-adapted to neurodivergent needs. The consistent themes of communication difficulties, sensory sensitivities, and previous traumatic experiences [28,29] suggest that many observed outcomes are less a function of neurodivergence itself and more a consequence of the mismatch between patient needs and standard care protocols.

Our findings indicate that neurodivergent participants were more likely to experience pelvic girdle pain and vaginal bleeding, and reported a pattern of nausea distinct in its persistence and intensity. The substantially elevated prevalence of anxiety and depression is consistent with existing literature [1,22]. In contrast, no significant group differences were observed for several obstetric conditions like gestational diabetes or preeclampsia; however, these null findings may be influenced by the study’s limited sample size and potential underdiagnosis within the neurodivergent cohort. Rather than dwelling on the statistical significance of each comparison, we posit that the collective results underscore a central thesis: the current model of care frequently fails this population.

A prime example is the disproportionately high rate of cesarean sections. This is unlikely to be a purely biological phenomenon. Instead, it appears to be a culmination of several systemic failings: communication challenges that increase labor stress, sensory sensitivities that make the delivery environment overwhelming, and a lack of provider training that leads to operative delivery being used as a risk-management strategy. Similarly, the near-universal decline of breastfeeding and the frequent postpartum separation from newborns point to a profound lack of tailored, anticipatory support for sensory and anxiety-related challenges.

The challenges toward a neurodiversity-informed obstetrical care identified are not intractable. They are addressable through deliberate, systemic adaptations. The prevalent communication difficulties and trauma-based behaviors [28,29] highlight that standard patient–provider interactions are insufficient. Healthcare professionals require specific training to adapt their communication, provide clear and predictable information, and foster a sense of psychological safety.

Furthermore, the labor environment itself must be reconfigured. Prolonged labor progression and high stress levels may be mitigated by environmental adjustments to reduce sensory overload, the provision of continuous one-to-one support, and clear, concise communication about labor progress. Such strategies can facilitate more efficient labor and reduce the perceived need for operative interventions.

On the basis of these findings, we developed a set of supportive strategies to guide healthcare professionals (Supplementary File S1). These recommendations focus on proactive, individualized approaches—including anticipatory guidance for breastfeeding, sensory accommodations, and structured communication aids—that can empower neurodivergent women and improve their care experiences [30].

In conclusion, the data compel a move away from a deficit-focused model that pathologizes neurodivergent traits. Instead, we must adopt a genuinely patient-centered framework that anticipates diverse needs and adapts the care system accordingly. Tailoring communication, respecting sensory sensitivities, and ensuring individualized support are not merely accommodations but essential components of ethical, effective, and equitable perinatal care for neurodivergent women. By rethinking care strategies to be neurodiversity-informed and trauma-conscious, we can align clinical practice with the ethical standards of modern medicine.

## 5. Conclusions

Collectively, these findings underscore the need for heightened awareness among healthcare professionals of the specific risks and challenges faced by pregnant women with NDs, including physical discomfort, mental health difficulties, and barriers to accessing care. Developing individualized care pathways, offering clear guidance on when to seek help, and creating supportive healthcare environments may improve outcomes and overall experiences for this population.

## 6. Limitations

Several limitations should be considered when interpreting the findings of this study. First, the sample size was relatively small, which limits statistical power and the generalizability of the results. Small numbers may also contribute to null findings for less frequent outcomes, such as certain pregnancy complications, and make it difficult to detect subtle differences between groups. Nevertheless, we consider this study was successfully conducted.

Second, accurately defining neurodivergent status in the perinatal context presents challenges. Diagnostic heterogeneity, variable reporting, and reliance on medical records or self-report may introduce misclassification or variability in the identification of neurodivergent participants. These factors complicate comparisons across studies and may influence observed associations between neurodivergent status and pregnancy or labor outcomes.

Finally, the complexity of perinatal care for neurodivergent women—encompassing communication difficulties, sensory sensitivities, mental health challenges, and individualized care needs—creates inherent variability in outcomes that is difficult to fully capture in retrospective analyses. Future studies with larger, more diverse samples, standardized diagnostic criteria, and prospective data collection are needed to better understand the unique needs of this population and to inform tailored, evidence-based maternity care practices.

## Figures and Tables

**Table 1:** Pregnancy conditions.

Complication	Control Group (*n* = 21)	Neurodivergent Group (*n* = 18)	OR (95% CI)	*p*-Value	*p*-Value (FDR Adj.)
Pelvic girdle pain	5 (23.8%)	12 (66.7%)	6.0 (1.5–24.2)	0.01	0.02
Vaginal bleeding	3 (14.3%)	8 (44.4%)	4.9 (1.1–21.7)	0.04	0.05
Gestational diabetes	2 (9.5%)	3 (16.7%)	1.9 (0.3–11.6)	0.64	0.72
High blood pressure	2 (9.5%)	3 (16.7%)	1.9 (0.3–11.6)	0.64	0.72
Preeclampsia	1 (4.8%)	1 (5.6%)	1.2 (0.07–19.7)	1.00	1.00
Eclampsia	0	0	–	–	–
Polyhydramnios	1 (4.8%)	2 (11.1%)	2.5 (0.2–29.7)	0.59	0.68
Placenta previa	1 (4.8%)	1 (5.6%)	1.2 (0.07–19.7)	1.00	1.00
Placental abruption	1 (4.8%)	1 (5.6%)	1.2 (0.07–19.7)	1.00	1.00
Hyperemesis gravidarum	2 (9.5%)	3 (16.7%)	1.9 (0.3–11.6)	0.64	0.72
Anxiety	4 (19.0%)	11 (61.1%)	6.6 (1.6–26.9)	0.007	0.01
Depression	3 (14.3%)	9 (50.0%)	6.3 (1.4–27.3)	0.02	0.03

**Table 2:** Frequency on nausea during pregnancy.

	Control Group (*n* = 21)	Neurodivergent Group (*n* = 18)	aOR (95% CI)	*p*-Value
Nausea			6.0 (1.30–27.77)	0.035
*n*	21	18		
Nausea every day, lasting throughout the day	3 (14.3%)	9 (50.0%)		
Nausea every day, not lasting throughout the day	4 (19.0%)	3 (16.7%)		
Nausea less frequently than every day	7 (33.3%)	4 (22.2%)		
No nausea during pregnancy	7 (33.3%)	2 (11.1%)		

**Table 3:** Attendance of prenatal appointments.

	Control Group (*n* = 21)	Neurodivergent Group (*n* = 18)
Attended all ultrasound appointments	20 (95.2%)	16 (88.9%)
Missed ≥1 ultrasound appointment	1 (4.8%)	2 (11.1%)
Attended all physician appointments	20 (95.2%)	12 (66.7%)
Missed ≥1 physician appointment	1 (4.8%)	6 (33.3%)

## Data Availability

The data presented in this study are available in the article and Supplementary Material.

## Supplementary Materials

The following supporting information can be downloaded at: https://www.mdpi.com/article/10.3390/jpm15110557/s1, Supplementary File S1: Supportive strategies for women with NDs receiving perinatal care.
