# Supplementary material for: Pregnancy and Childbirth in Neurodivergent Women: Shift Towards Personalized Maternity Care
Source: J Pers Med. 2025 Nov 17;15(11):557. doi: 10.3390/jpm15110557 (PMC12653929; doi:10.3390/jpm15110557)
Supplement: Supplementary file 1 [file jpm-15-00557-s001.zip › jpm-3913021-supplementary.pdf]

Supplements

Supportive strategies for women with NDs receiving perinatal care

Planning and Individualized Care

1. Prenatal Care Planning: Work collaboratively with the woman to develop a detailed, individualized birth plan, rather than designing it on her behalf. This document should be a live, adaptable record of her preferences, sensory sensitivities, communication styles, and triggers. This aligns with the core principles of patient-centered care and shared decision-making, which are crucial for vulnerable populations.
2. Antenatal Consultations: Offer pre-admission tours of the labor and delivery unit to familiarize the patient with the environment. Schedule dedicated, longer prenatal appointments to build rapport, discuss procedures in detail, and reduce anxiety about the unknown. This strategy helps to desensitize patients to the clinical environment and builds trust.

Sensory Environment Modifications

1. Sensory Aids and Adjustments: Actively promote and facilitate the use of sensory regulation tools. This includes allowing and encouraging the use of noise-canceling headphones, tinted glasses, or a sleep mask to manage auditory and visual overstimulation. Provide access to low-light settings and quiet, private rooms whenever possible.
2. Sensory-Informed Procedures: Modify standard procedures to minimize distress. For example, warm the ultrasound gel, use a plastic speculum instead of a cold metal one, and minimize unnecessary physical touch. Always announce touch before initiating it. This acknowledges the high prevalence of sensory processing differences in autistic individuals and others with NDs.

Communication Adaptations

1. Clear, Concrete, and Concise Language: Use direct, unambiguous language. Avoid metaphors, sarcasm, or abstract instructions. Offer information in written, pictorial, or easy-read formats alongside verbal explanations to aid comprehension and retention.
2. Explaining the "Why": Prior to any procedure, explain not only *what* will happen but also *why* it is necessary, how long it will take, and what sensations she might experience. This reduces anxiety stemming from unpredictability and supports autonomous decision-making.

3. Respecting Communication Styles: Identify and respect her preferred method of communication. Some patients may need more time to process questions and formulate responses. Avoid interrupting or finishing her sentences. For non-speaking individuals or those who may shut down under stress, establish alternative communication methods in advance (e.g., communication cards, tablets, or pre-agreed gestures).

Supporting Autonomy and Reducing Anxiety

1. Trauma-Informed and Positive Reinforcement: Employ a trauma-informed care lens, recognizing that behaviors are a form of communication. Acknowledge distress, validate feelings, and offer choices to restore a sense of control. Provide positive, specific praise for coping and participation.
2. Consistent and Predictable Care: Strive for continuity of caregiver to build a trusting relationship and avoid the stress of repeating her history and needs to new staff. When change is unavoidable, ensure a thorough handover that includes her care plan.
3. Designated Support Person: Actively encourage and integrate a trusted support person (partner, doula, family member, friend) who understands her needs and can act as a communication bridge and advocate.

Interdisciplinary Collaboration and Staff Training

1. Specialist Liaison: Facilitate collaboration with the patient's existing mental health providers, neurologists, or ND specialists to coordinate care and manage co-occurring conditions effectively.
2. Mandatory Training: Implement mandatory training for all perinatal staff on the presentation of NDs in women, including concepts like masking, sensory processing differences, and communication styles. Training should focus on reducing diagnostic overshadowing—where new symptoms are incorrectly attributed to the existing ND—and combating implicit bias.
